# Supplementary figures and images for: SIRT4 as a novel interactor and candidate suppressor of C-RAF kinase in MAPK signaling
Source: Life Sci Alliance. 2024 Mar 18;7(6):e202302507. doi: 10.26508/lsa.202302507 (PMC10948936; doi:10.26508/lsa.202302507)

Repeat 1

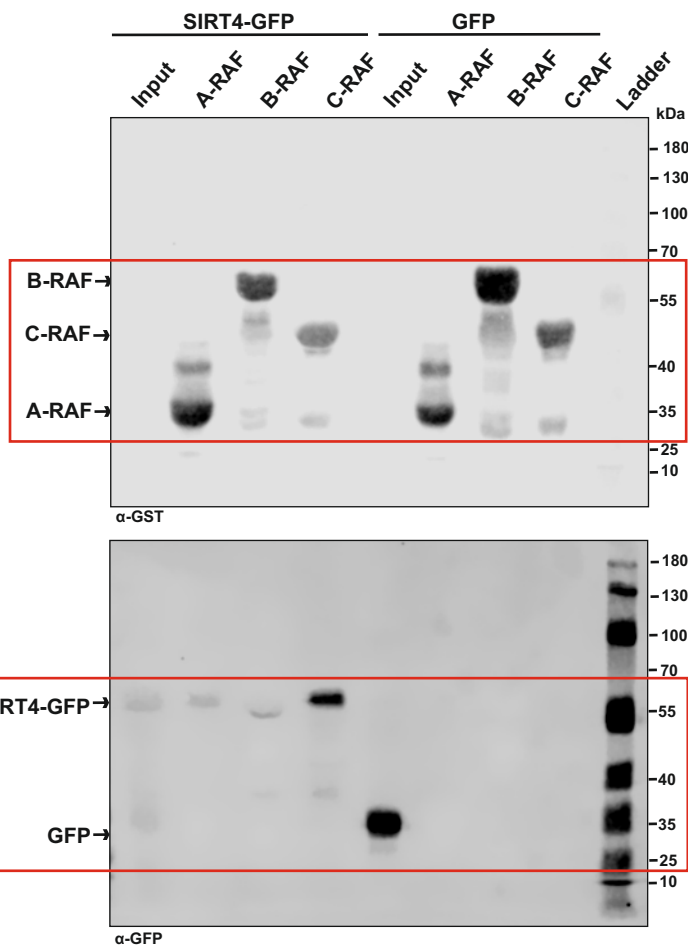

Repeat 2

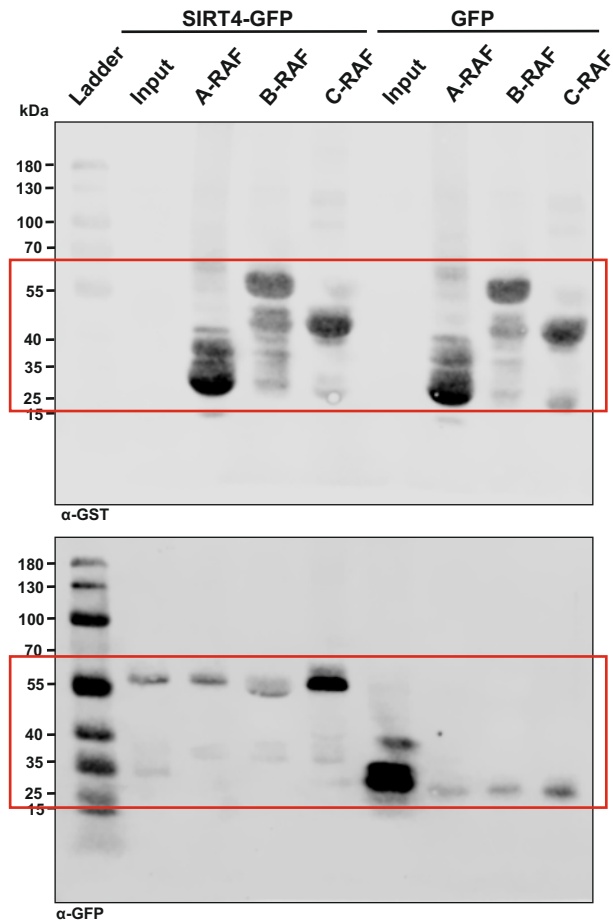

Repeat 3

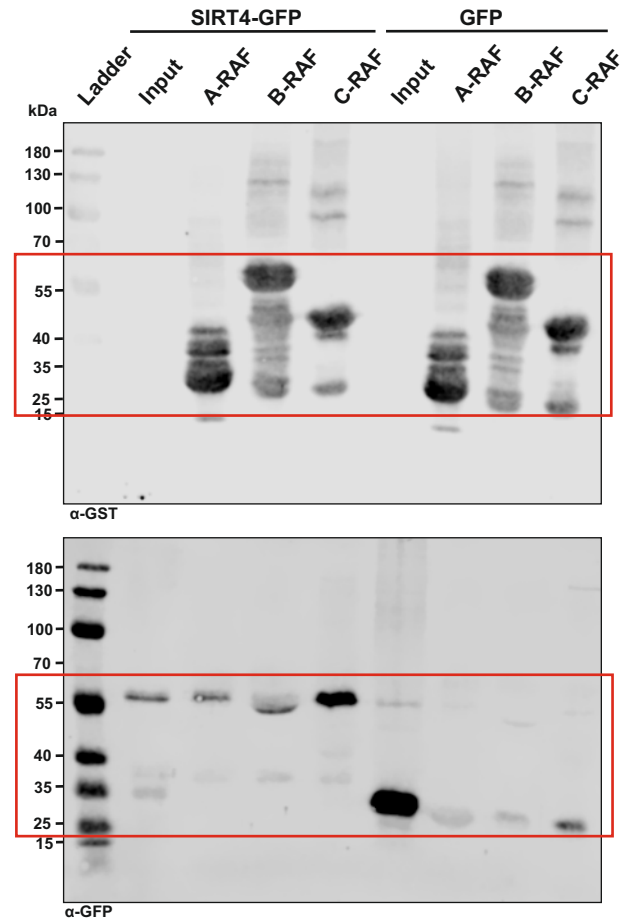

Supplement: Supplementary file 1 [file LSA-2023-02507_SdataF1.1.pdf]

Repeat 1

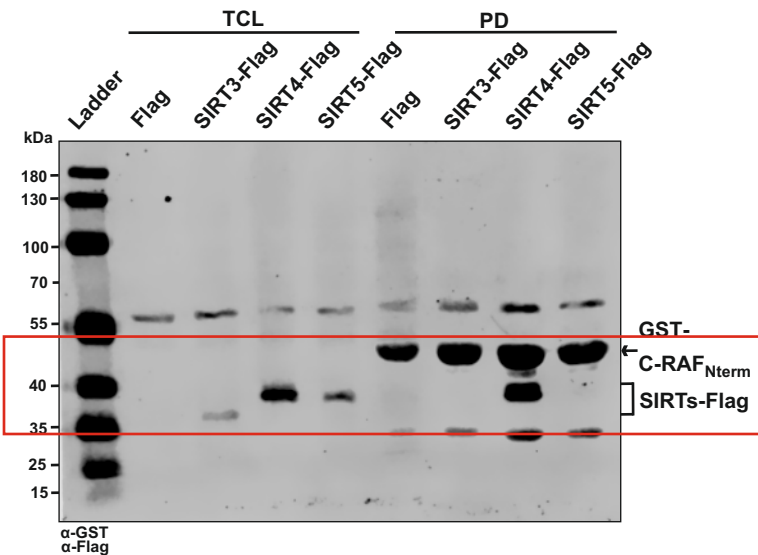

Repeat 2

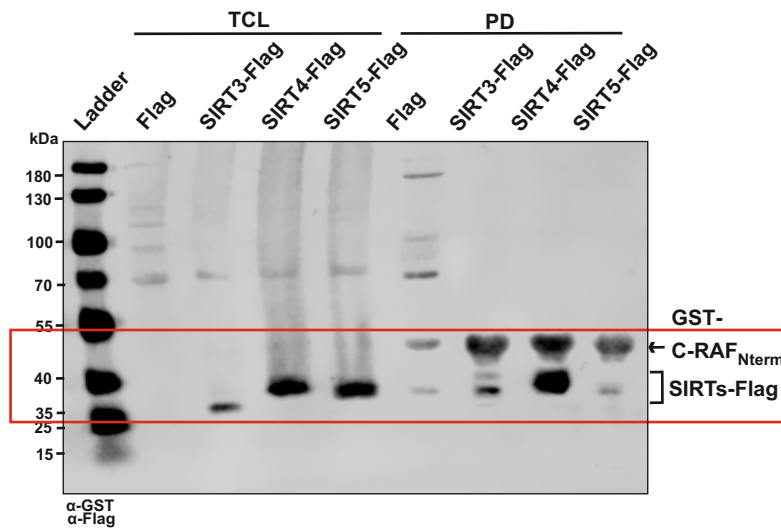

Repeat 3

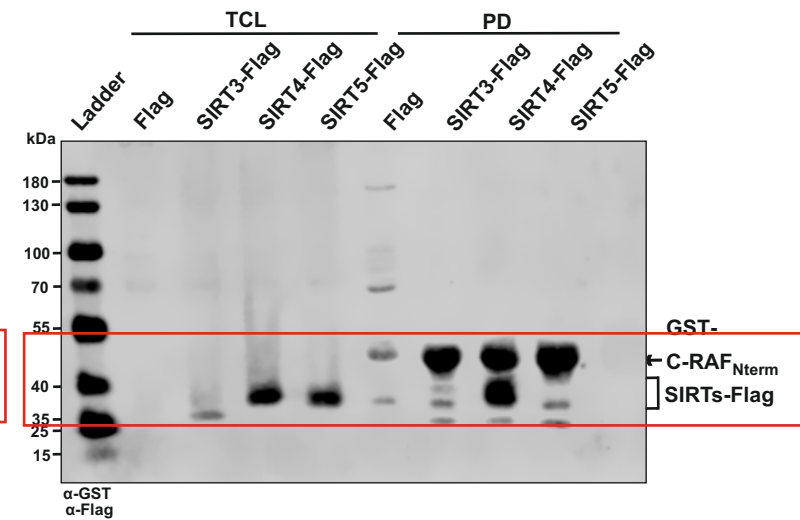

Supplement: Supplementary file 2 [file LSA-2023-02507_SdataF1.2.pdf]

Repeat 1

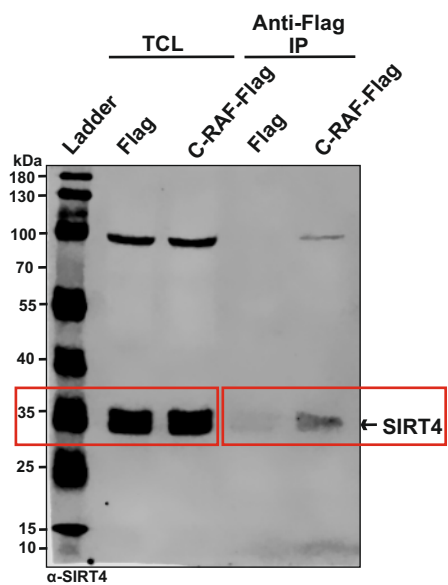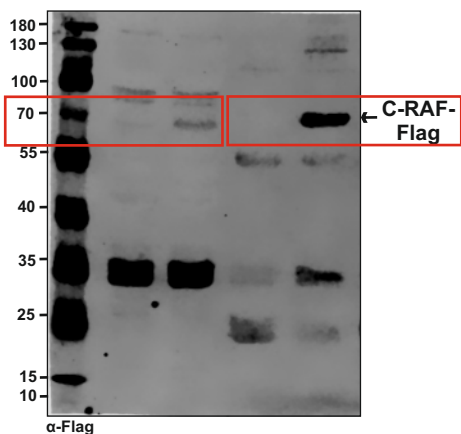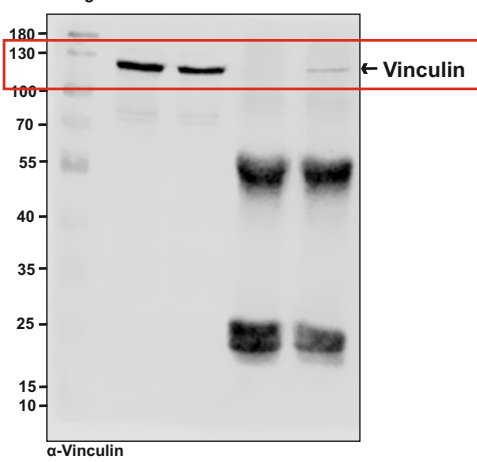

Repeat 2

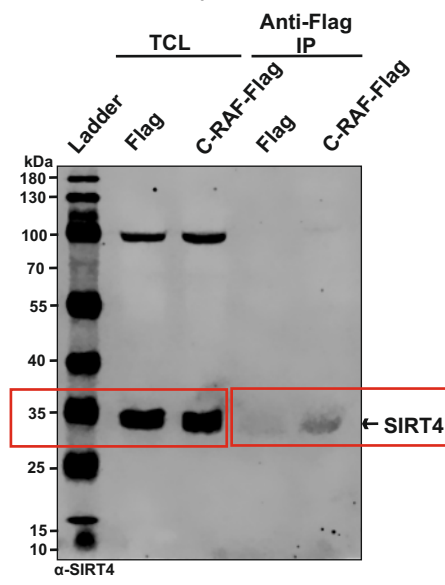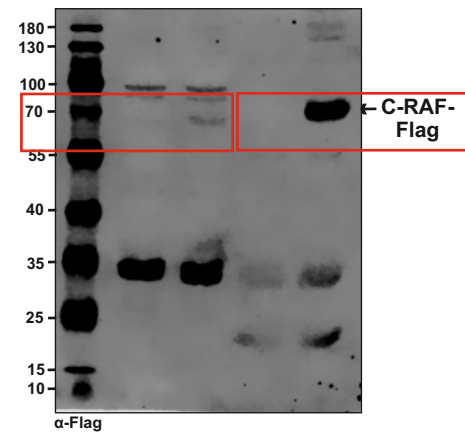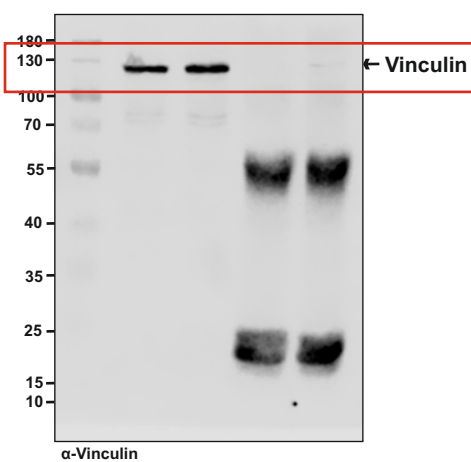

Repeat 3

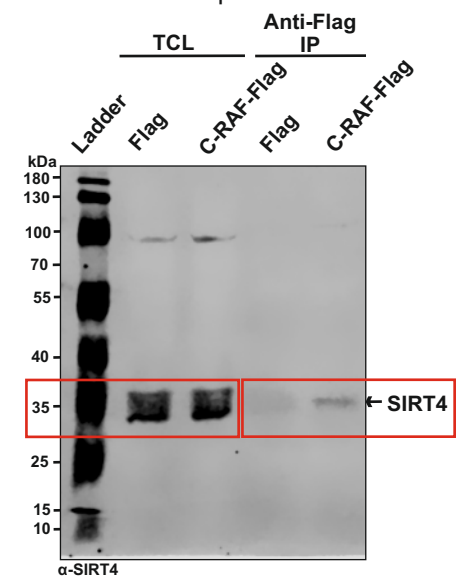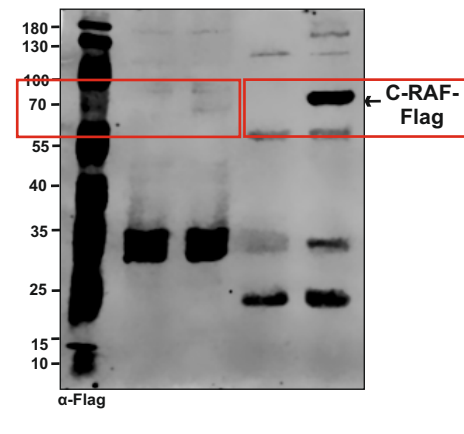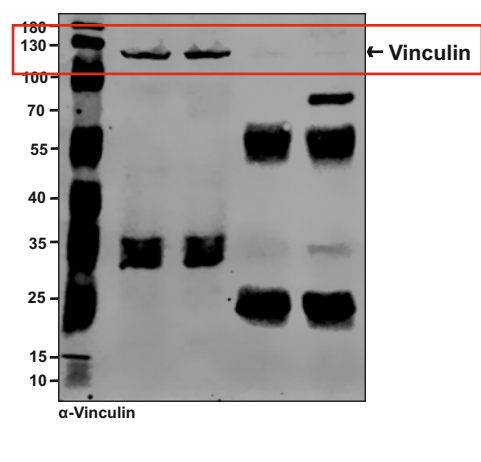

Supplement: Supplementary file 3 [file LSA-2023-02507_SdataF1.3.pdf]

Repeat 1

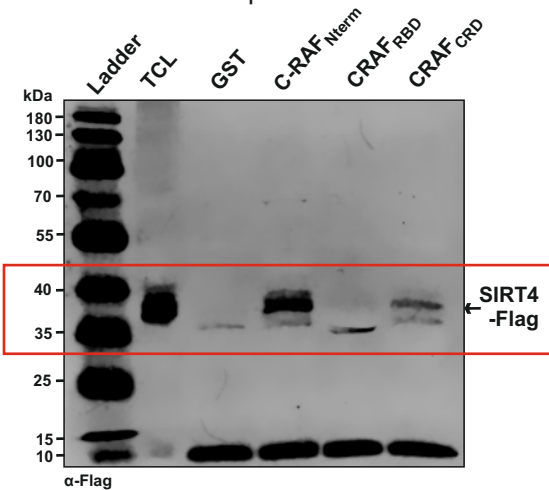

Repeat 2

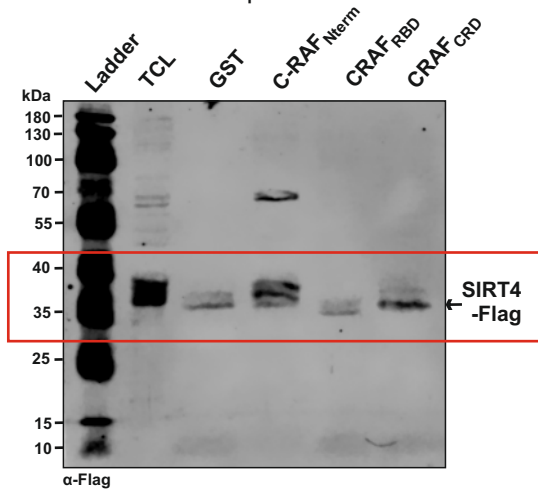

Repeat 3

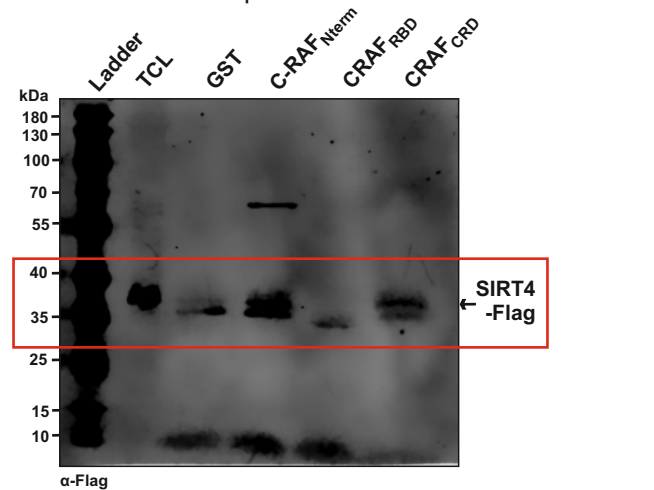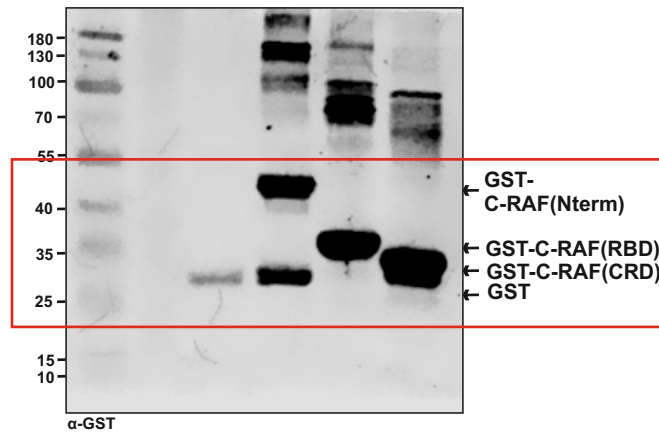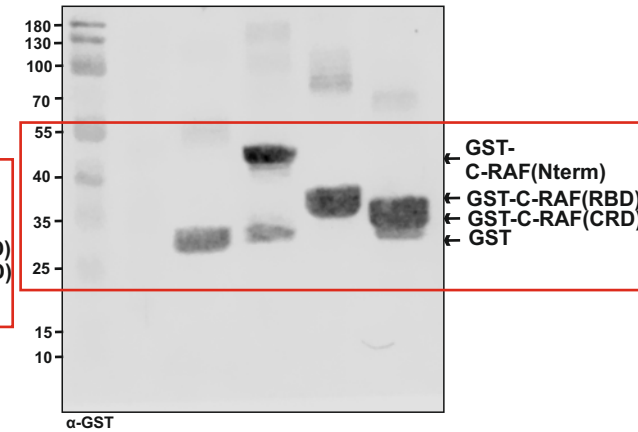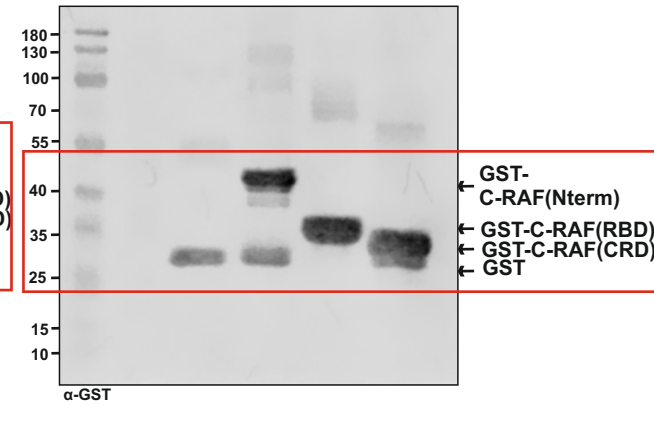

Supplement: Supplementary file 4 [file LSA-2023-02507_SdataFS3.pdf]

Repeat 1

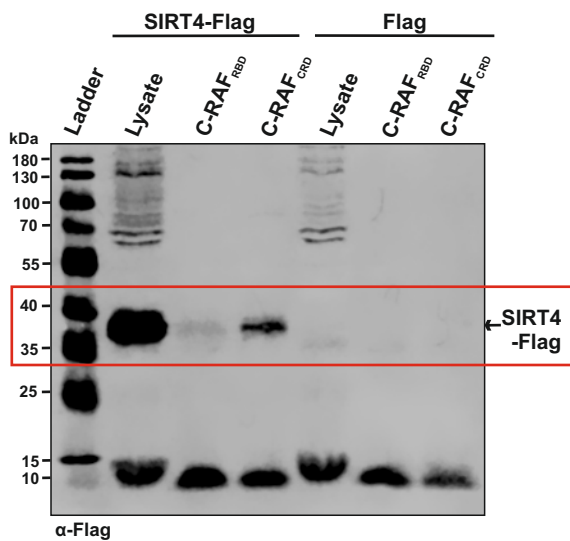

Repeat 2

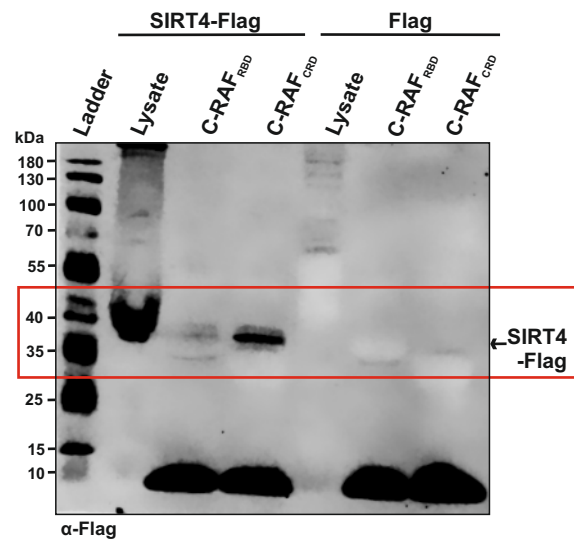

Repeat 3

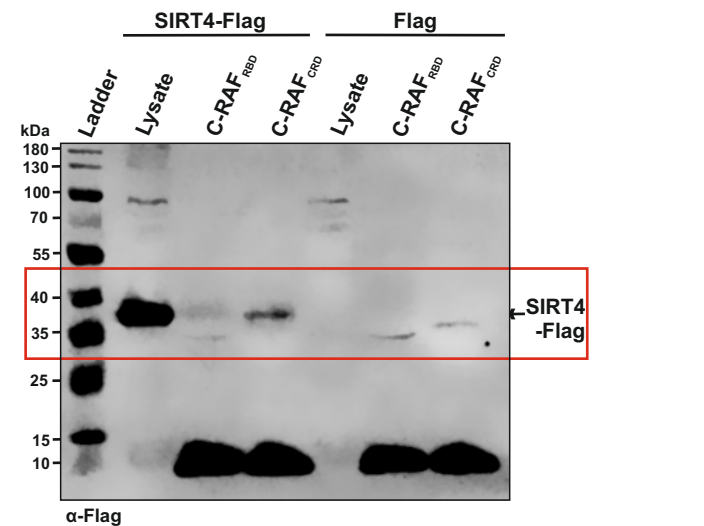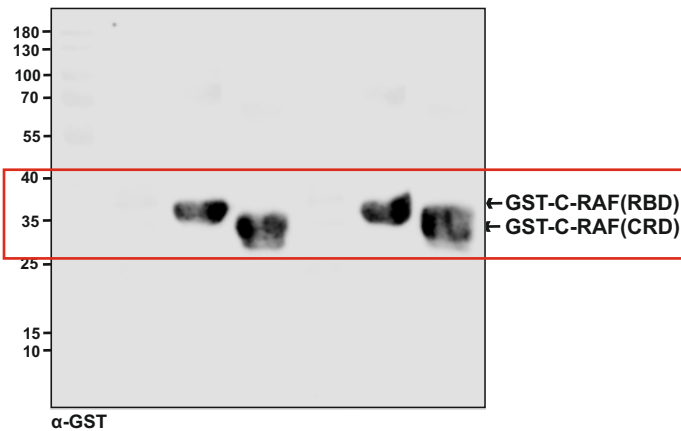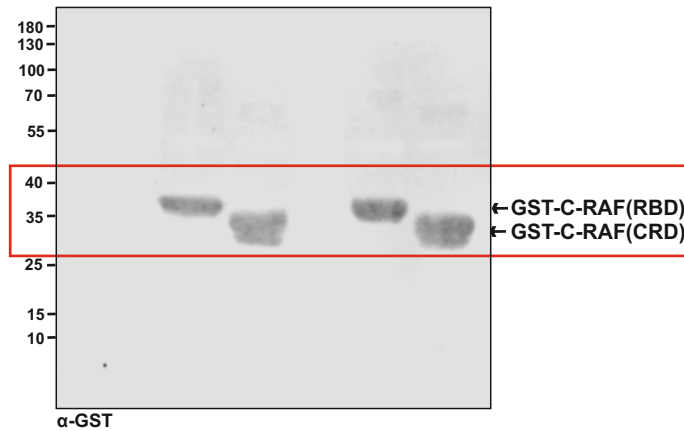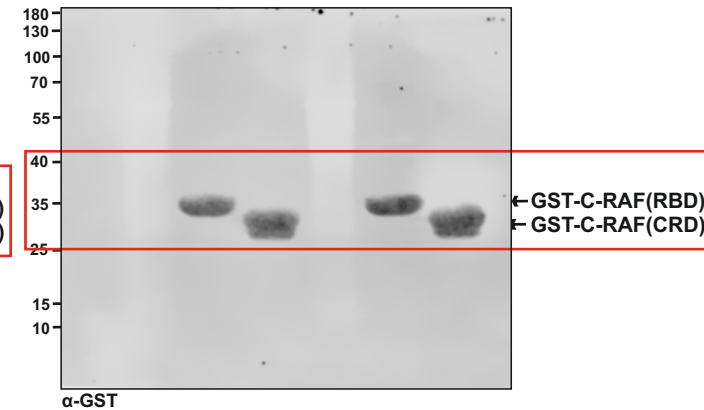

Supplement: Supplementary file 5 [file LSA-2023-02507_SdataF2.1.pdf]

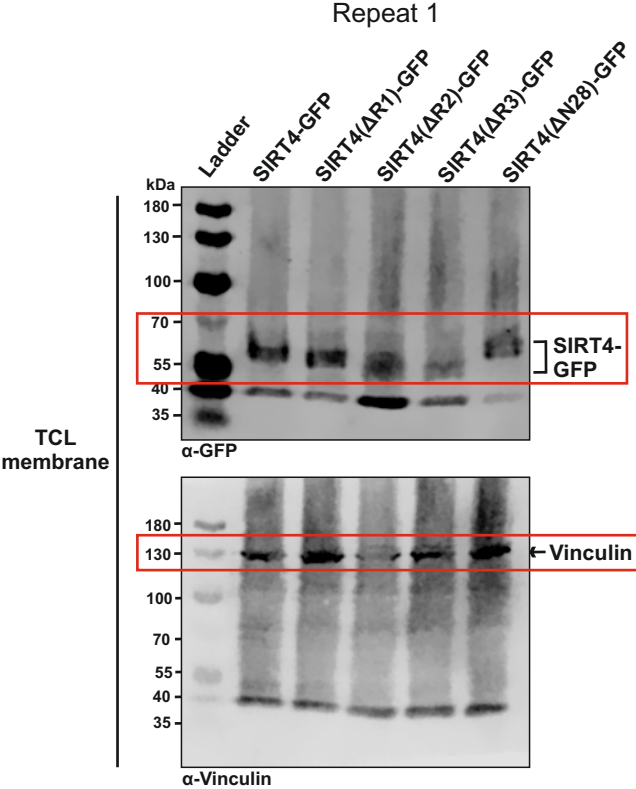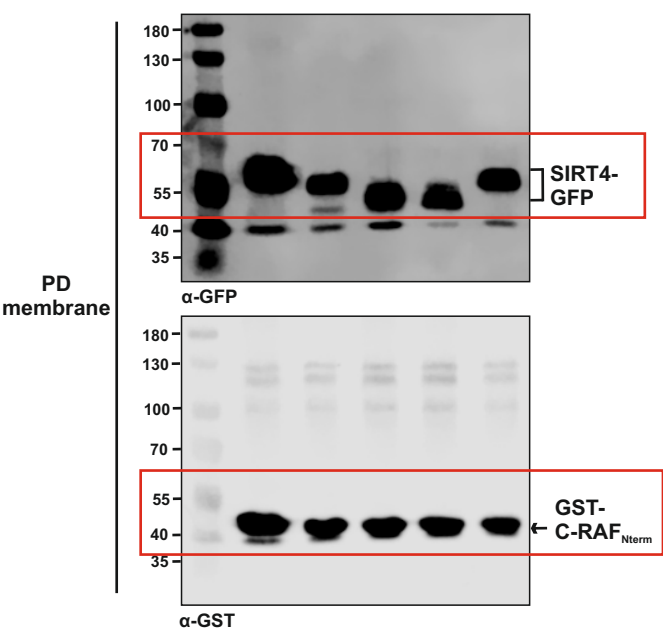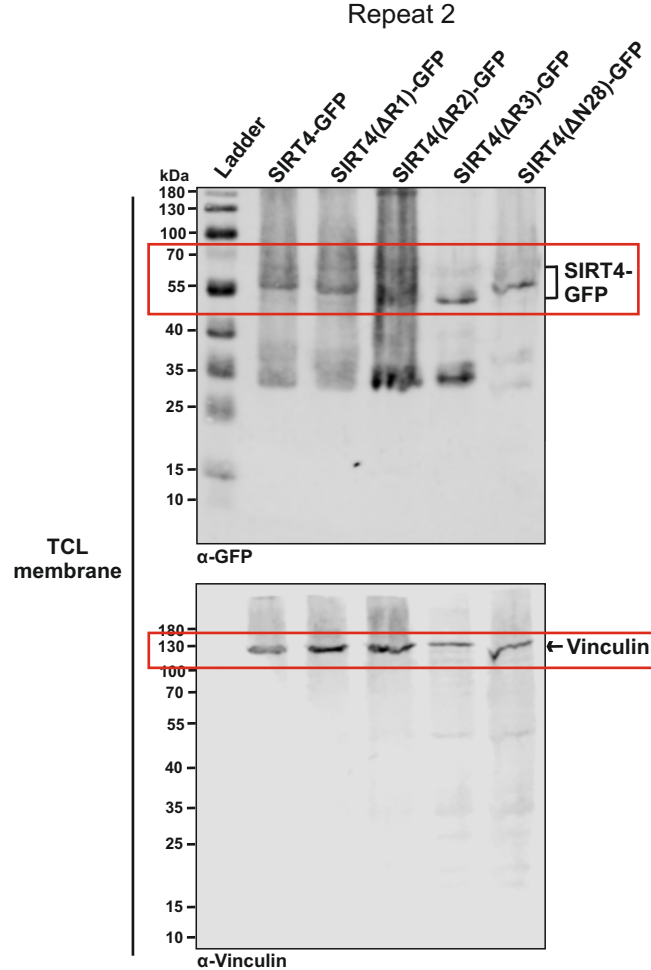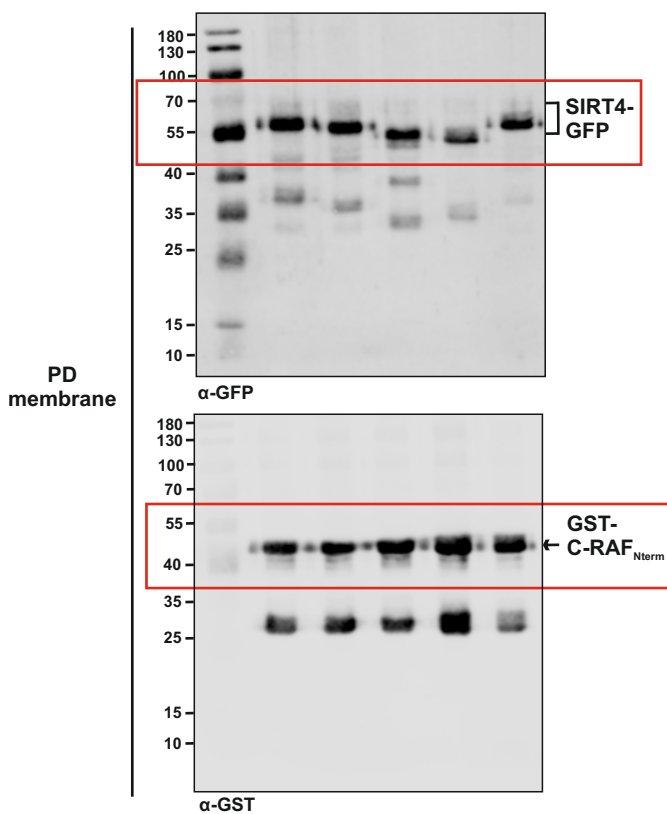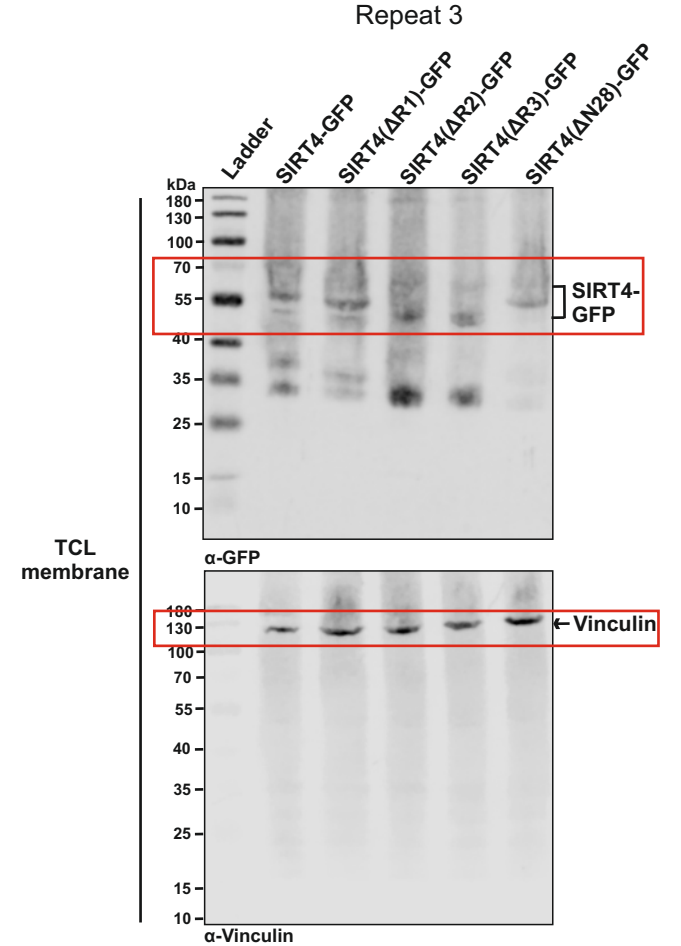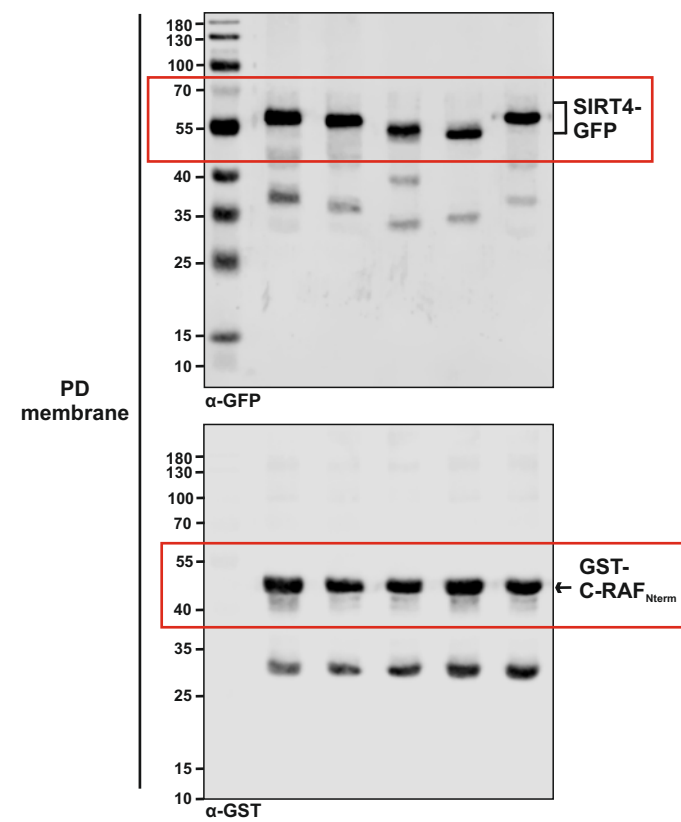

Supplement: Supplementary file 6 [file LSA-2023-02507_SdataF2.2.pdf]

Repeat 1

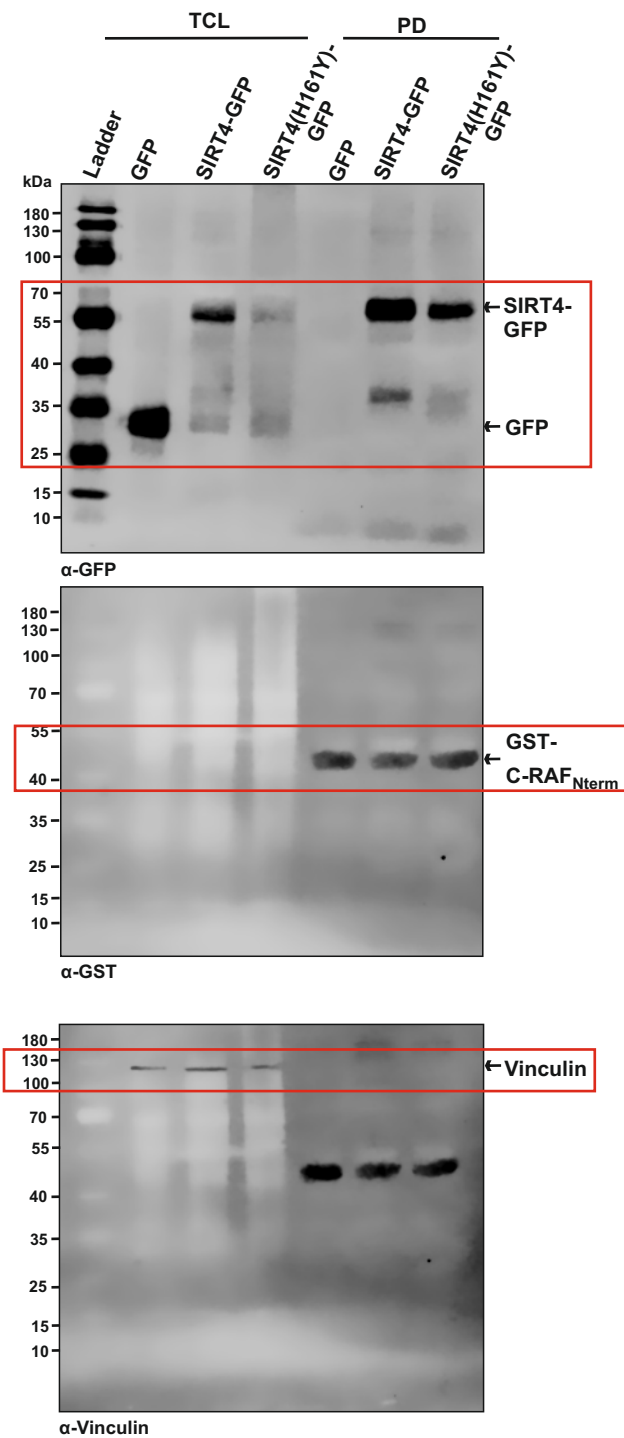

Repeat 2

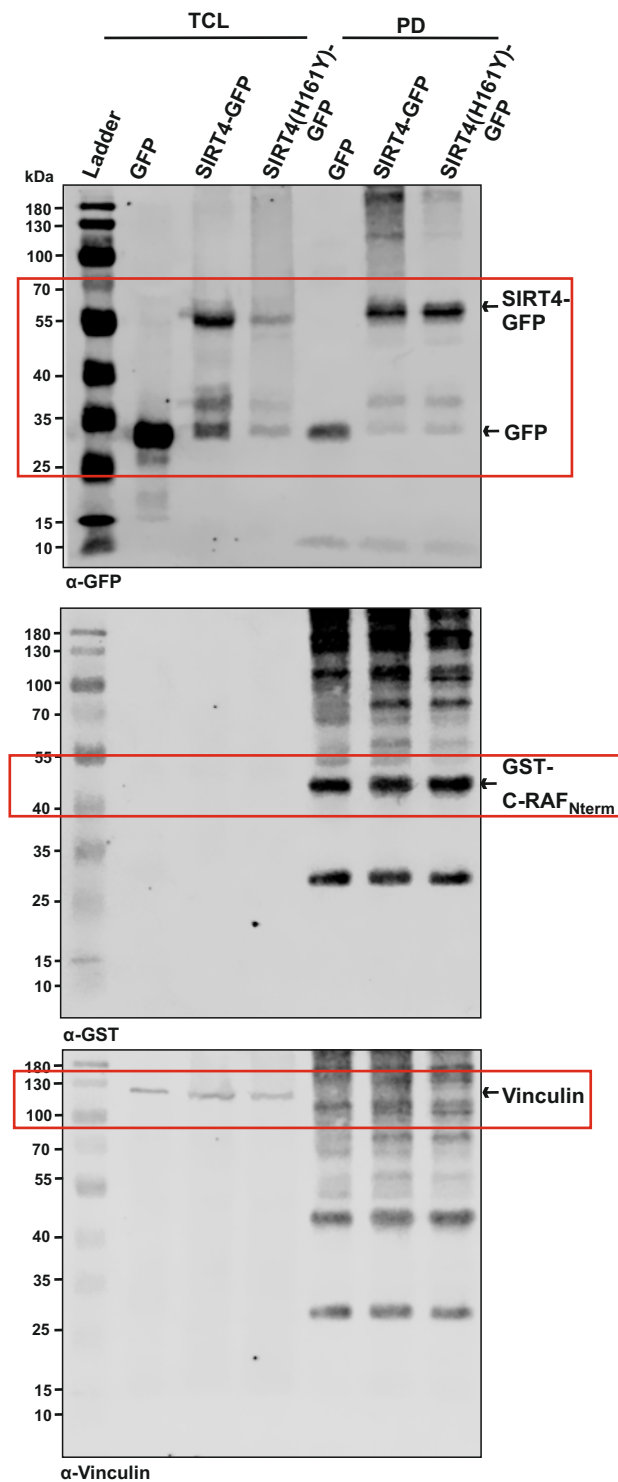

Repeat 3

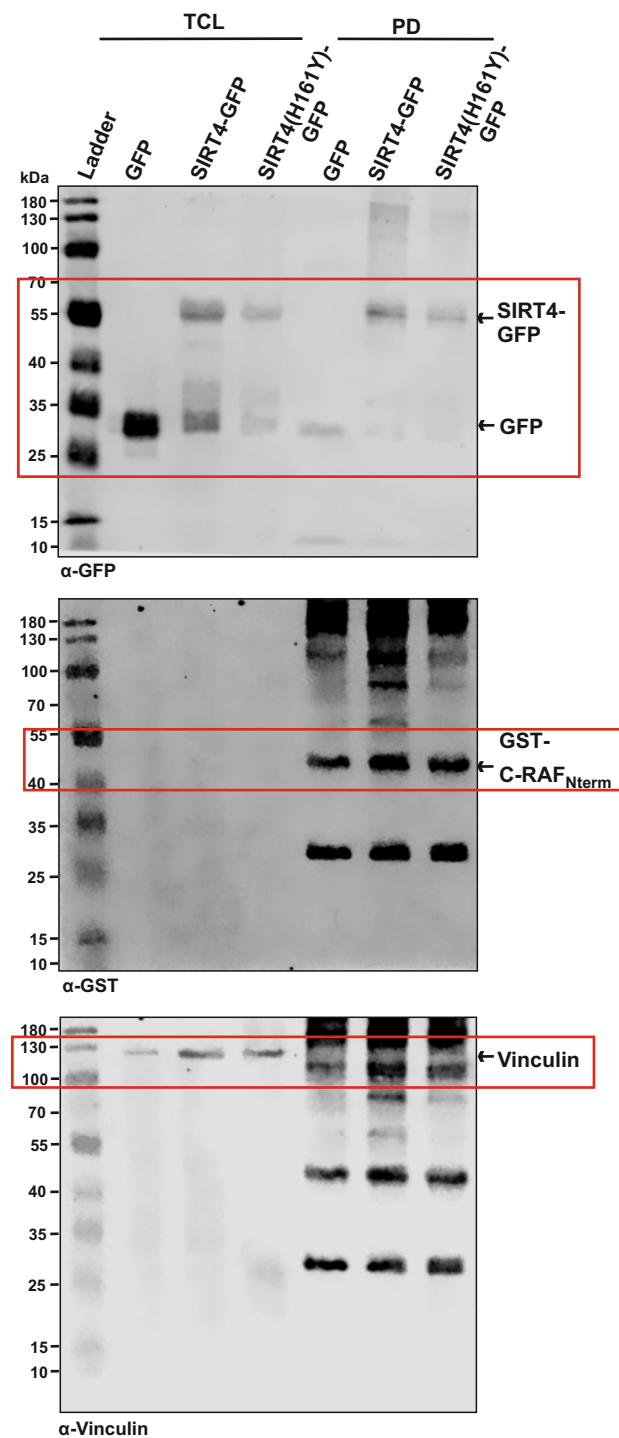

Supplement: Supplementary file 7 [file LSA-2023-02507_SdataFS5.pdf]

Repeat 1

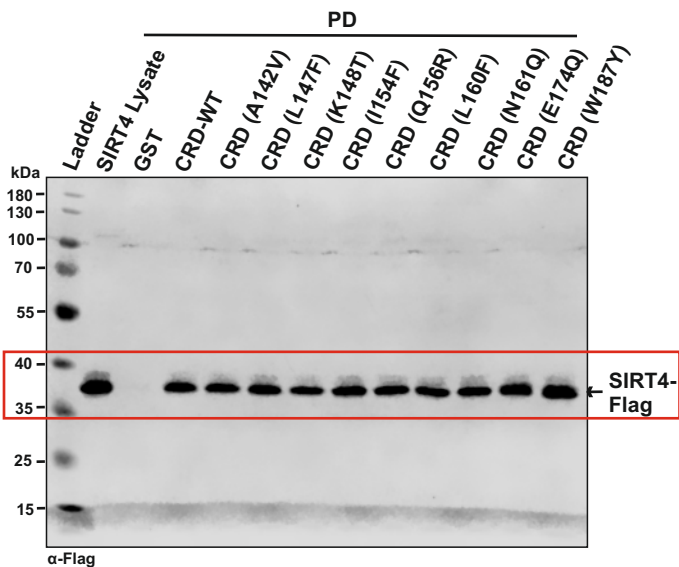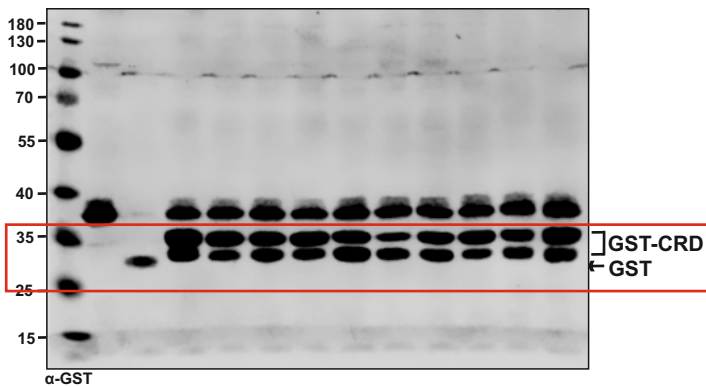

Repeat 2

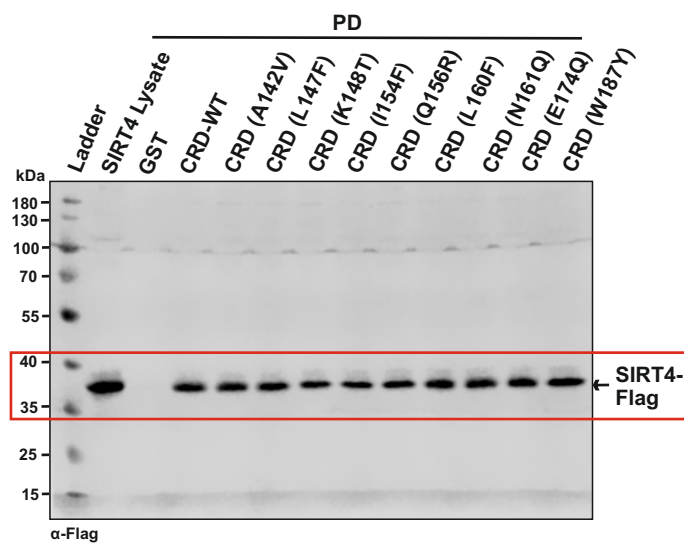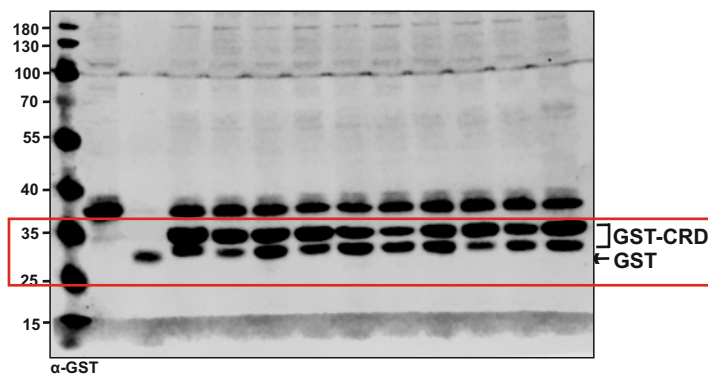

Repeat 3

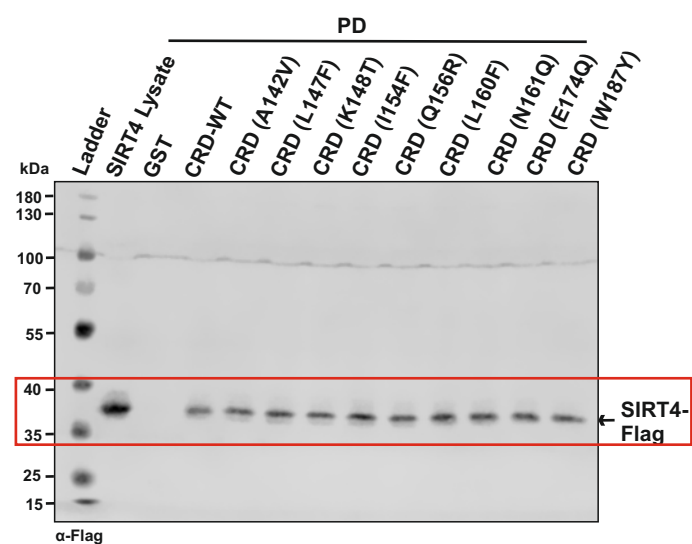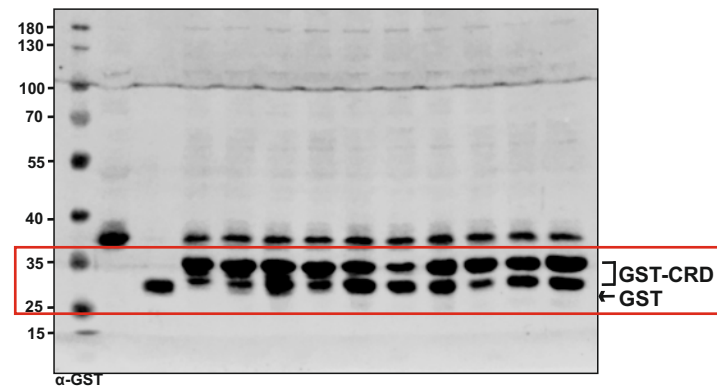

Supplement: Supplementary file 8 [file LSA-2023-02507_SdataF3.1.pdf]

Repeat 1

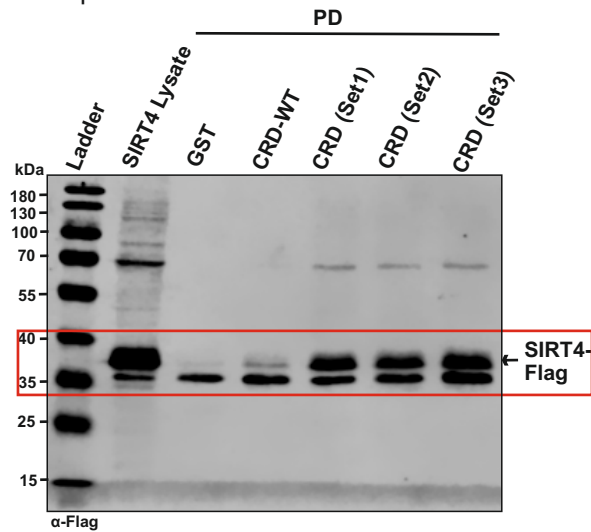

Repeat 2

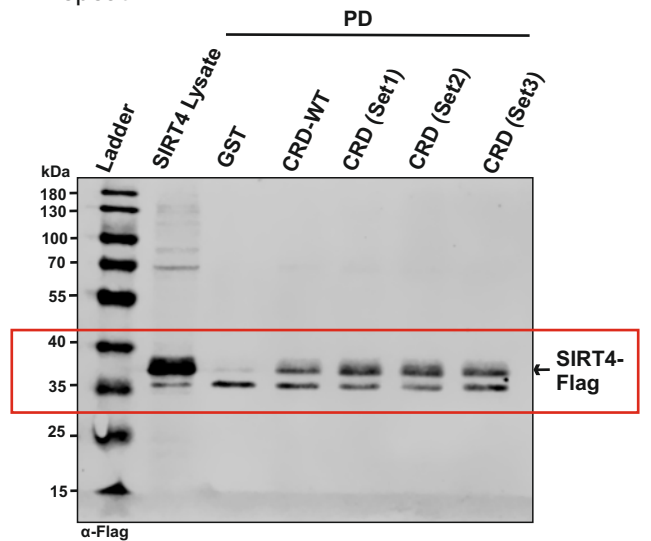

Repeat 3

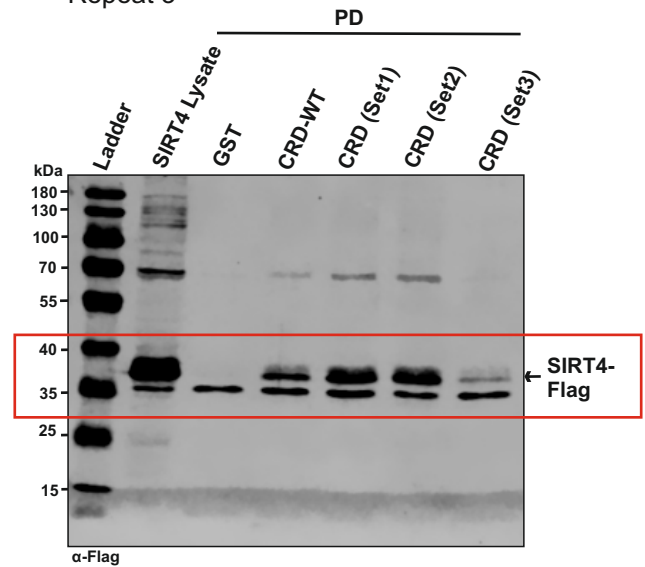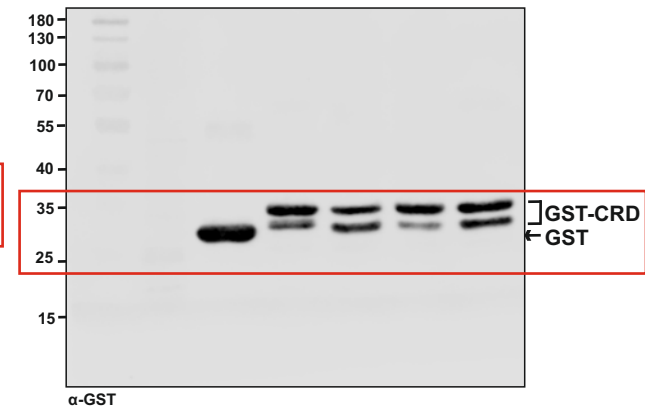

Supplement: Supplementary file 9 [file LSA-2023-02507_SdataF3.2.pdf]

# Repeat 1

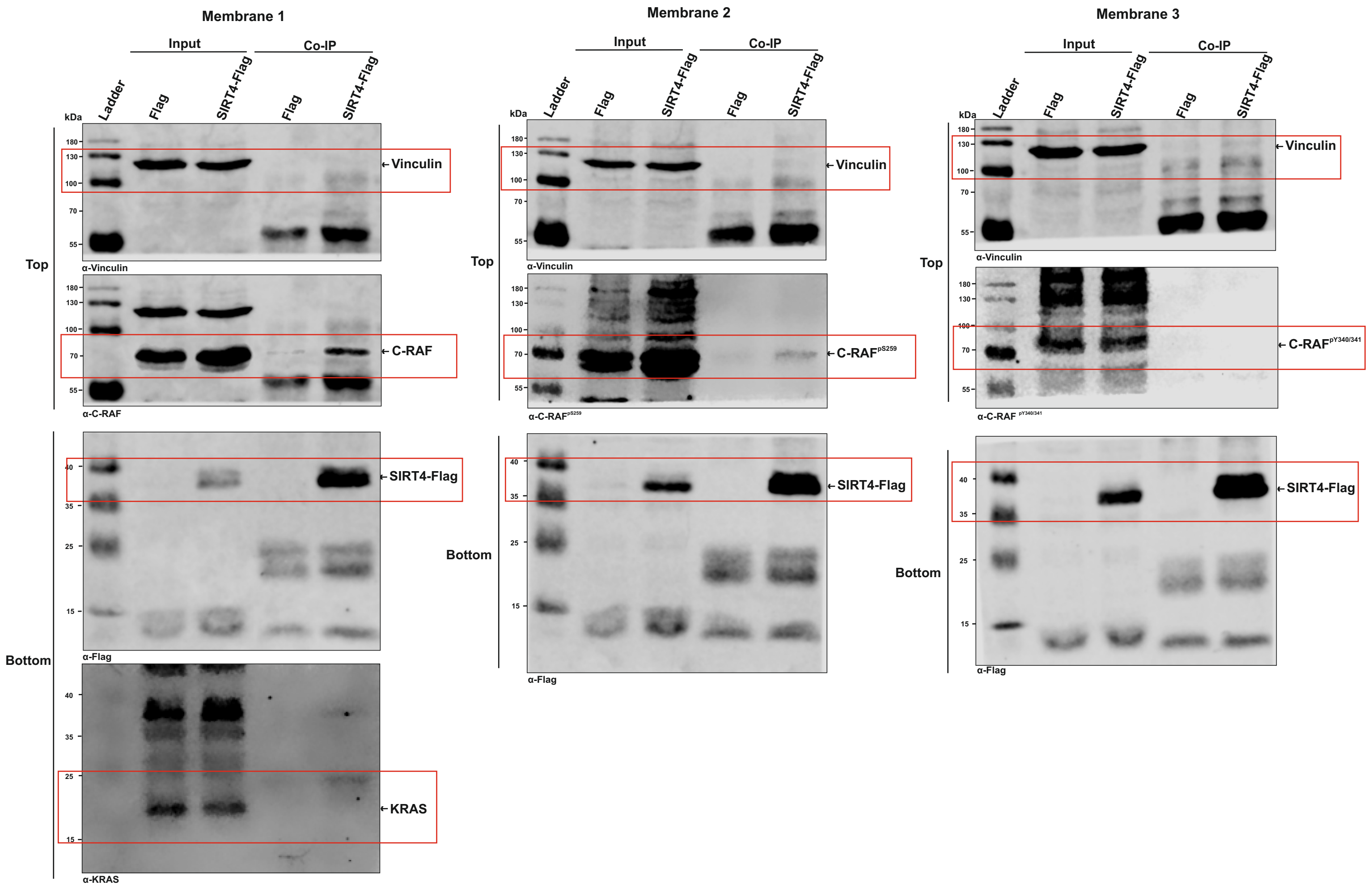

# Repeat 2

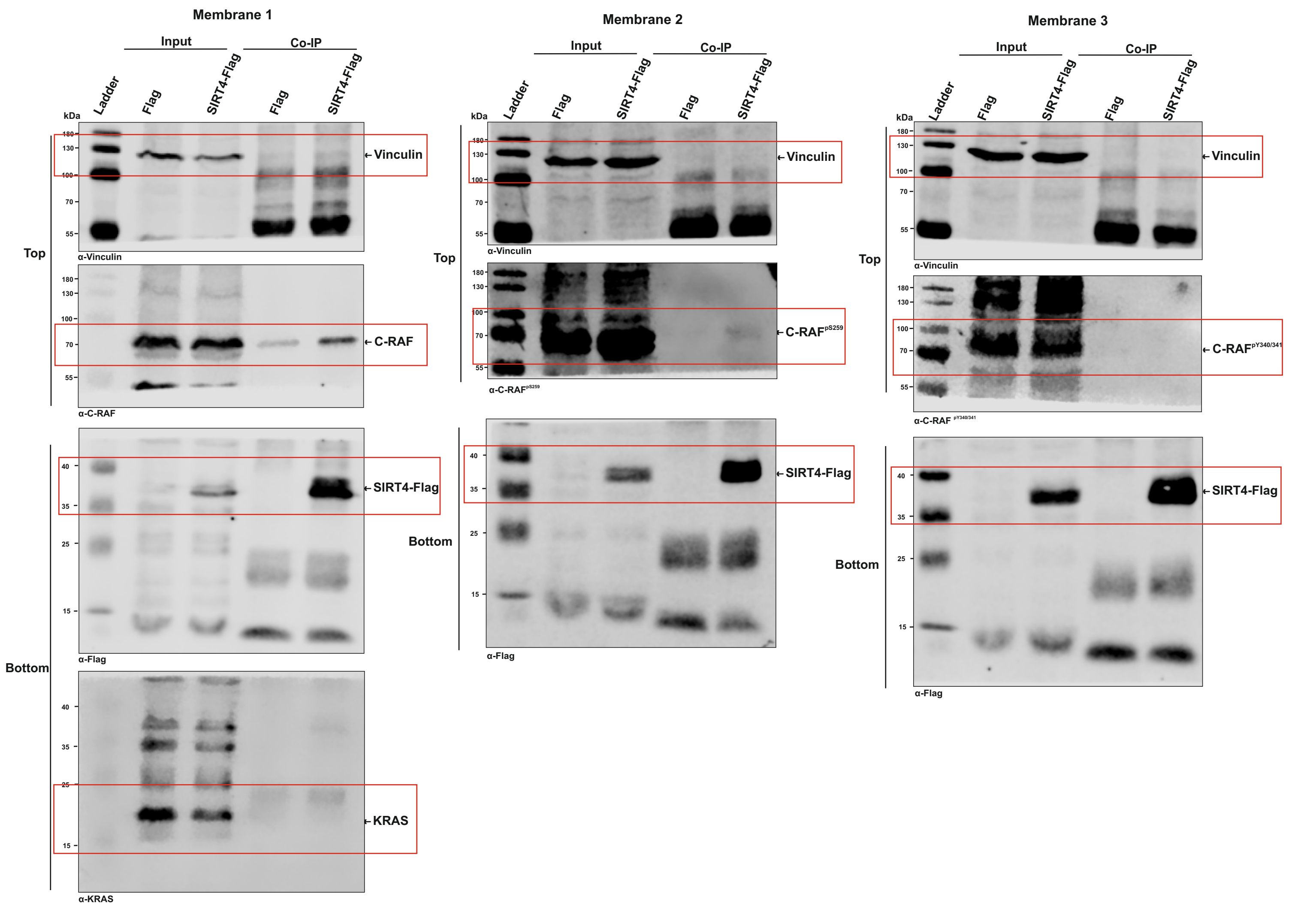

# Repeat 3

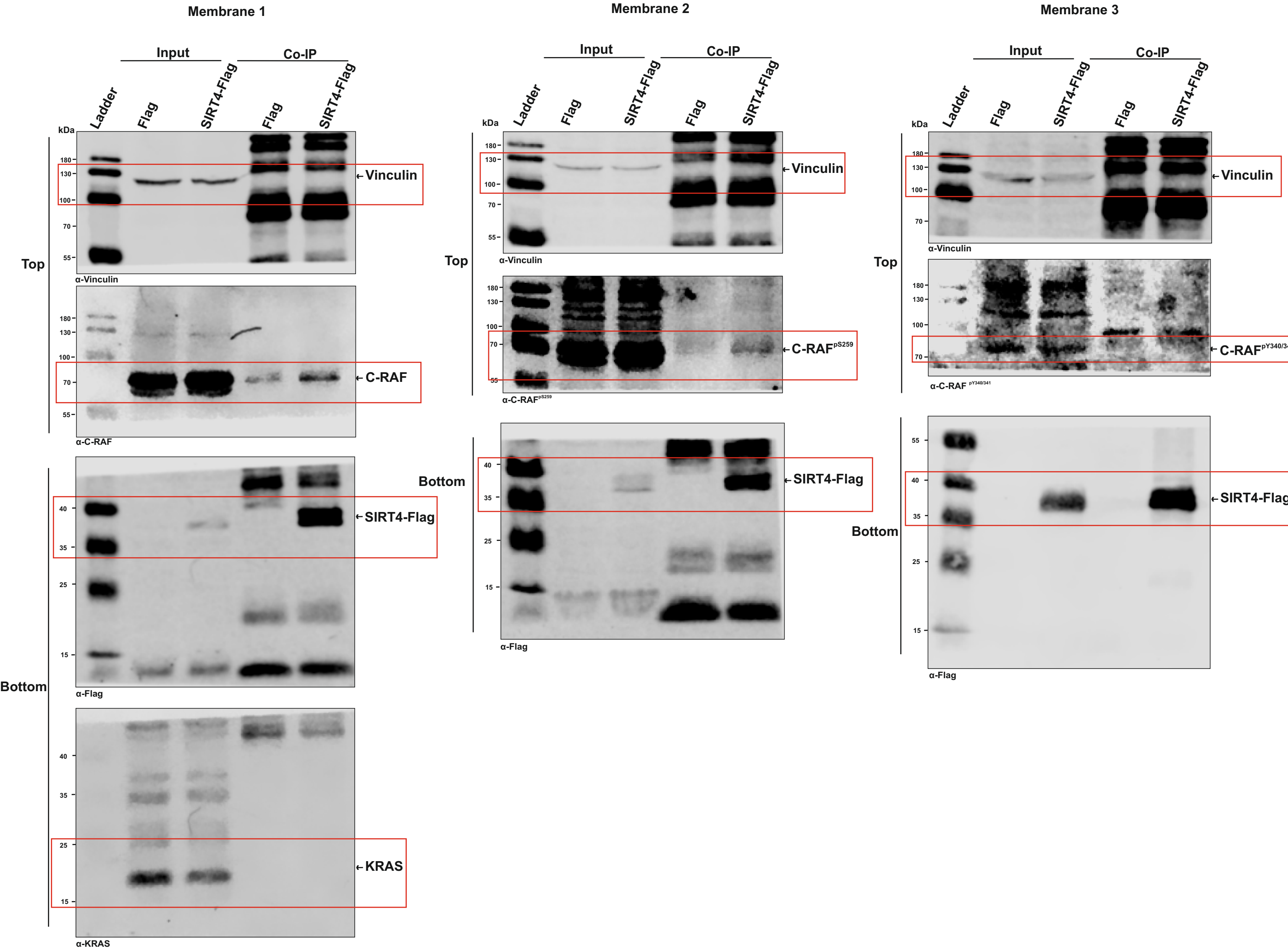

Supplement: Supplementary file 12 [file LSA-2023-02507_SdataF4.1.pdf]

## Repeat 1

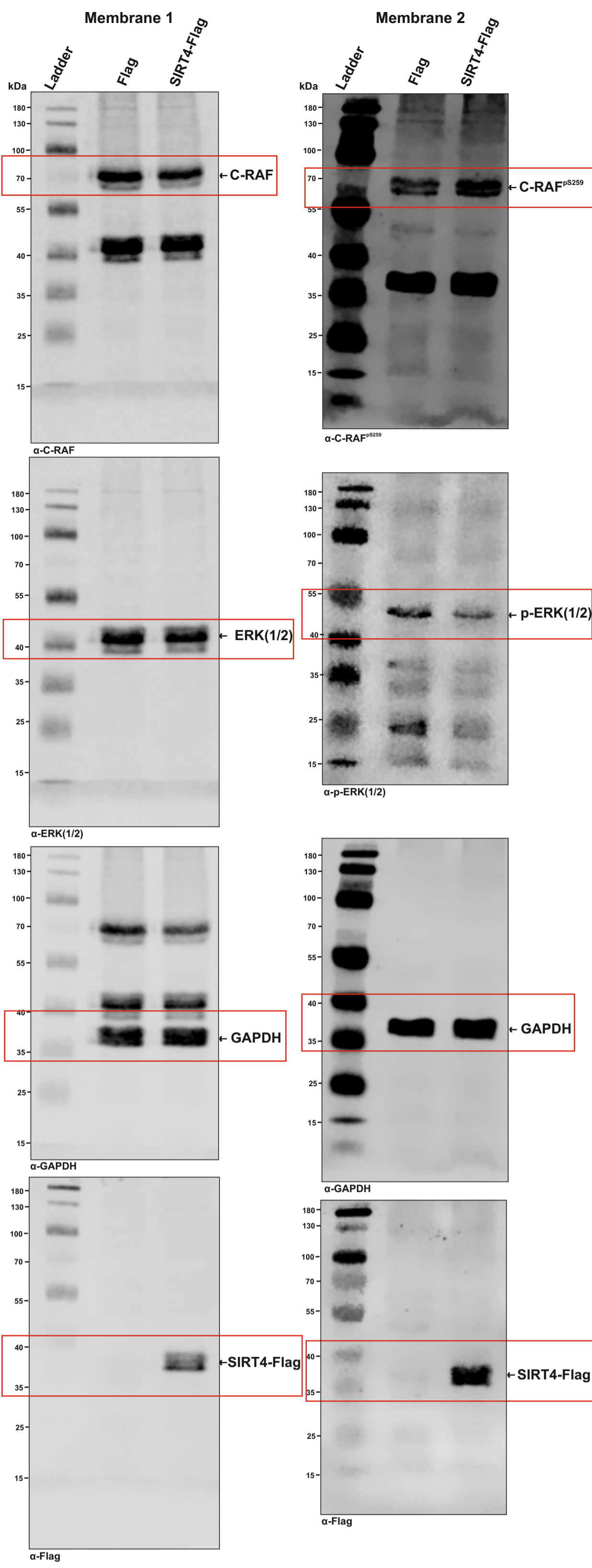

## Repeat 2

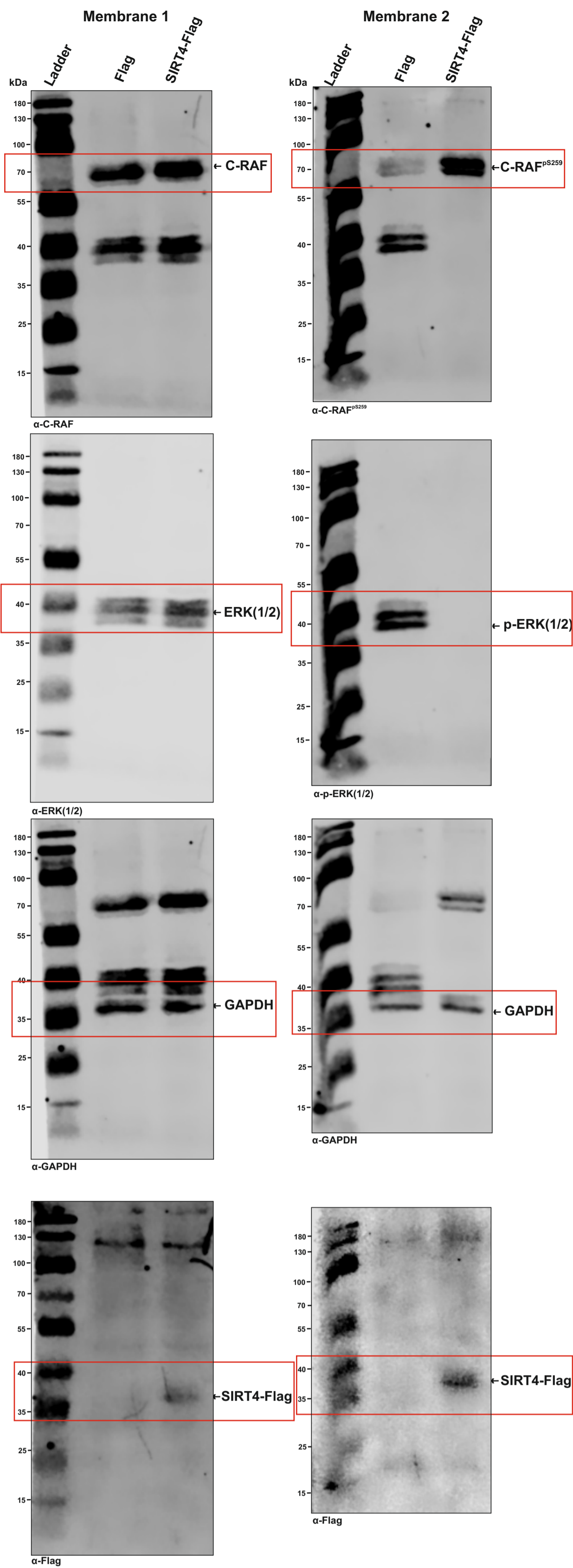

Repeat 3

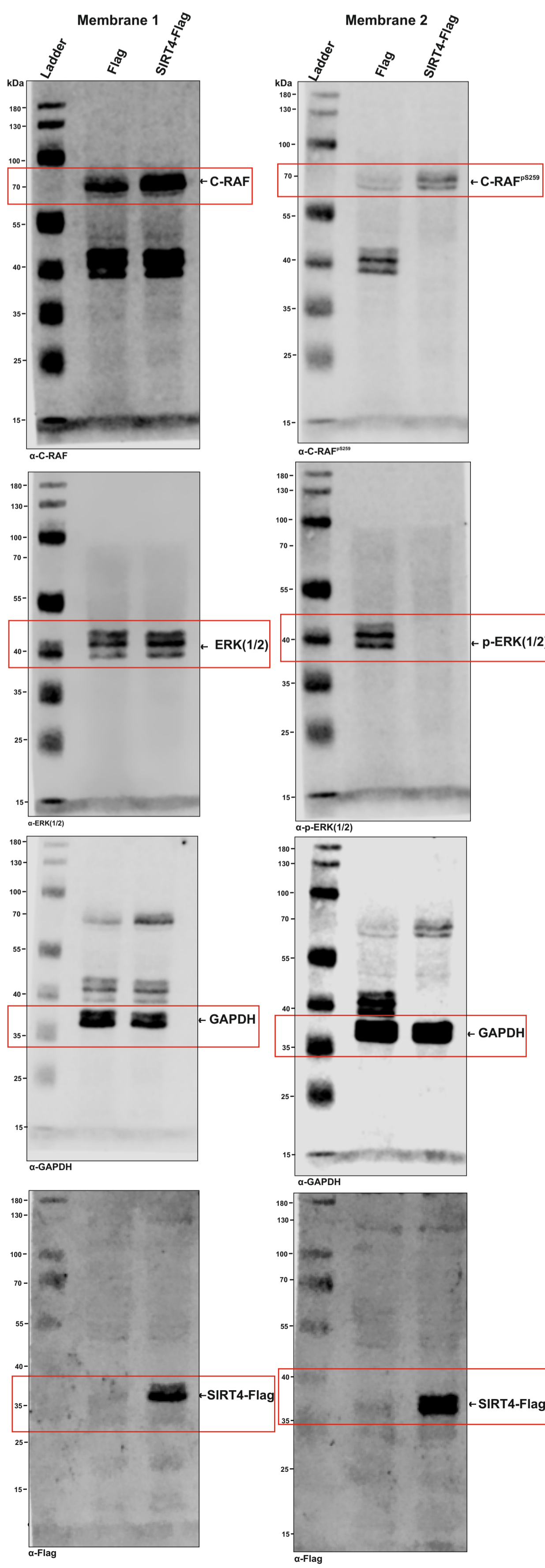

Repeat 4

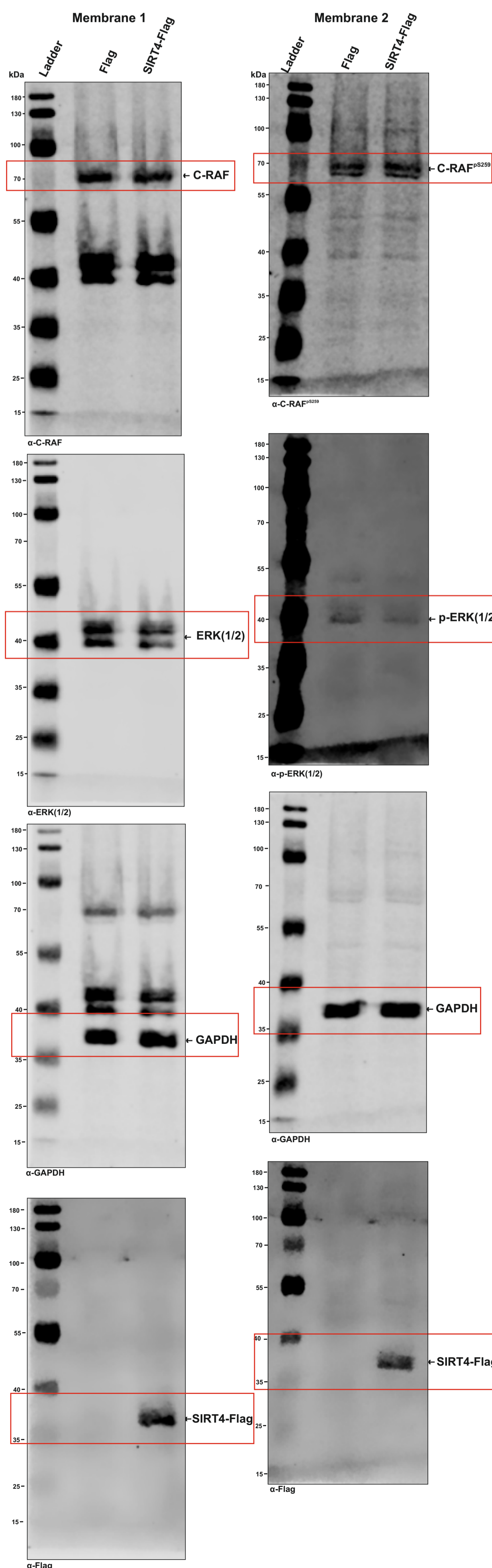

Supplement: Supplementary file 13 [file LSA-2023-02507_SdataF4.2.pdf]
